# Chitosan/Montmorillonite/Graphene Oxide Composites for the Adsorption of Dyes from Single-Component Solutions, Binary Mixtures, and Real Textile Wastewaters: On Understanding the Adsorption Interactions

**DOI:** 10.1021/acs.langmuir.5c03148

**Published:** 2025-08-19

**Authors:** Anastasia D. Meretoudi, Ioannis Kalampogias, Ioanna Koumentakou, Ioannis K. Kalavrouziotis, Athanasia K. Tolkou, George Z. Kyzas

**Affiliations:** † Hephaestus Laboratory, School of Chemistry, Faculty of Sciences, 37791 Democritus University of Thrace, GR 65404 Kavala, Greece; ‡ School of Science and Technology, 68992 Hellenic Open University, GR 23635 Patras, Greece

## Abstract

Industrial waste remains a global issue threatening human health and the balance of the ecosystem. Organic dyes are one of the most significant and widely found pollutants in wastewater. In this study, two novel composites, chitosan/montmorillonite (CS/MT) and CS/MT/graphene oxide (CS/MT/GO), were synthesized for wastewater management by adsorption of reactive black 5 (RB5) and methylene blue (MB) from aqueous solutions, the binary dye system, and real wastewater samples from the textile industry. Moreover, tripolyphosphate sodium (TPP) was used as a cross-linking agent for the bead’s formation during the synthetic process. The materials were extensively characterized using Fourier transform infrared spectroscopy (FTIR), scanning electron microscopy (SEM), Brunauer–Emmett–Teller (BET), and X-ray diffraction (XRD) techniques. SEM images revealed that the CS/MT/GO composite beads exhibited a larger diameter and a rougher surface than CS/MT, while BET shows that the surface area of CS/MT/GO (44.61 m^2^/g) is more extended than that of CS/MT (20.01 m^2^/g). The adsorption experiments showed that CS/MT/GO effectively removed RB5 and MB, providing 222.35 and 203.98 mg/g of adsorption capacities, respectively. Notably, the removal efficiency for RB5 reached 99.1% at pH 2, while, for MB, a maximum removal efficiency of 80.29% was observed at pH 12. Furthermore, the results fit the Freundlich isotherm and pseudo-second-order (PSO) kinetic models well. Moreover, thermodynamic analysis revealed that both dyes’ adsorption processes were endothermic and spontaneous. This study aimed to synthesize a reusable GO biomaterial that would be effective in both anionic and cationic dyes. Additionally, reusability was studied, and it was found that CS/MT/GO is more suitable for removing RB5 than MB after 5 regeneration cycles. However, there appears to be a competitive effect between RB5 and MB in binary systems. Finally, the CS/MT and CS/MT/GO composites are suitable for industrial applications, demonstrating excellent adsorption capacity, stability, and reusability for effectively removing pollutants from wastewater.

## Graphical abstract

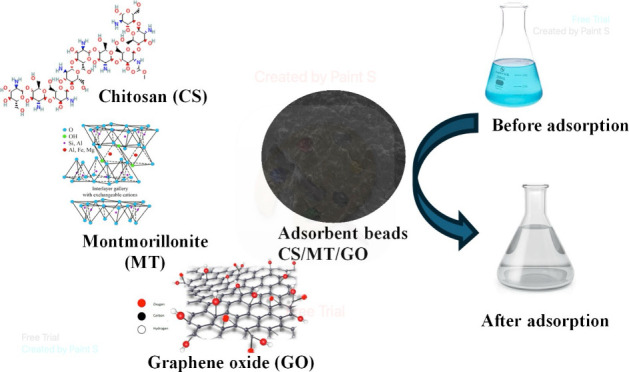

## Introduction

The dyeing industry is a significant sector directly linked to various human activities, including textiles, decoration, food, hair coloring, etc. According to the European Environment Agency, the textile sector is the third largest worldwide industry contributing to environmental pollution.^1^ However, synthetic dyes are more widely used than natural dyes. Approximately 800 000 tons are produced globally per year, while 18% of it is in wastewater, posing a threat to flora and fauna. Moreover, synthetic dyes are considered toxic because of their complex structure and the existence of heavy metals, such as Zn, Cu, Pb, Cr, and Co, in their chain.^2^ In addition, the slow biodegradation of dyes is a significant challenge, as the lack of effective removal methods allows these pollutants to accumulate in aquifers over time, eventually reaching the food chain and affecting human health. Consequently, exposure to pollutants can hurt human health, causing respiratory problems, poisoning, or even carcinogenesis.^3^ Azo dyes, such as reactive black 5 (RB5) and acid blue 113 (AB113), represent one of the largest and most significant classes of synthetic dyes. Their defining characteristic is the presence of one or more azo groups (−N=N−), which act as molecular bridges connecting two organic moieties, typically with at least one being aromatic.^4^ A common and simple example of such a compound is azobenzene. Due to their structural features, many azo dyes are intensely colored, making them widely used in manufacturing paints, inks, and textiles.^5^ Among industrial pollutants, synthetic dyes pose a significant environmental and health concern due to their extensive use, persistence, and potential to cause long-term effects, such as eye irritation, dermatitis, and allergic reactions.^6^

This study focuses primarily on RB5 and methylene blue (MB), which refer to anionic and cationic synthetic dyes, respectively. Therefore, RB5 has been considered carcinogenic and toxic to aquatic organisms and MB. However, they are used for medical purposes in small doses; they can cause several side effects at higher concentrations, such as poisoning and blood damage. However, releasing these dyes into aquatic systems can seriously impact the ecosystem, reduce biodiversity, and slow down the natural water purification process. In recent decades, the awareness of their management has been raised, and the scientific community is trying to find cost-effective ways to manage the water pollution from wastewater.^7^

Many methods for wastewater management have been developed, such as coagulation/flocculation or sedimentation, filtration, disinfection, biosorption,^8^ or ion exchange.^9^ This study examines adsorption to remove RB5 and MB from even authentic (textiles) wastewaters. Adsorption is considered a more cost-effective and environmentally friendly method than others, i.e., membrane filtration.^10^ Furthermore, sorption is one of the most frequently used methods for wastewater treatment due to its low cost,^11^ high removal efficiency (with up to 99.9% removal of dye pollutants), and ease of applicability. Among adsorption’s benefits, it is noteworthy that the adsorbent materials can be reused for many cycles, reducing the operational cost significantly while promoting a sense of recycling, which is a crucial step forward for the industrial sector. The literature reports various adsorbent materials used for environmental applications, such as natural polymers (chitin, chitosan, cellulose, etc.), clays (kaolin, montmorillonite, etc.), and carbon-based materials (graphene oxide, activated carbon, etc.).^12^

Chitosan (CS) is a natural linear polysaccharide composed of distributed β-(1→4)-linked d-glucosamine (deacetylated unit) and a *N*-acetyl-d-glucosamine biopolymer. It is derived from various natural sources, such as the cell walls of fungi, crustaceans, shellfish, and insect exoskeletons.^13^

Due to the amino group, CS interacts with pollutants, such as heavy metals, organic, or pharmaceutical compounds, via electrostatic interactions or hydrogen bonds.^14^ Moreover, it is known that CS is a biodegradable and nontoxic natural polysaccharide, which is environmentally friendly and suitable. Further, the adsorption capacity of CS depends on various factors, including pH, temperature, and pollutant concentration. To increase the adsorption properties of CS, scientists combine CS with other adsorptive materials, particularly clay. The attention of the scientific community is focused on clay minerals, such as kaolinite, montmorillonite, smectite, illite, and chlorite, as they represent affordable, efficient, and easily accessible materials for the removal of dyes, offering a promising solution to environmental challenges.^15^ Montmorillonite (MT) is a physical clay with high cation-exchange capacity (CEC) and a porous internal structure.^16^ Additionally, MT excels in binding nutrients, pollutants, and heavy metals, making it invaluable in agriculture and environmental remediation applications.^17^ The chemical formula of MT is (Si_4_)­(Al_2–*y*_Mg_*y*_)­O_10_(OH)_2*y*_M^+^·*n*H_2_O, where M^+^ signifies a monovalent cation and *y* is the degree of isomorphous substitution. Moreover, MT’s solid structure imparts to the material well swelling properties and mechanical properties. Thus, montmorillonite interacts covalently with the polysaccharide chains of chitosan, forming a three-dimensional structure that is suitable for wastewater treatment.

Additionally, graphene oxide (GO) is a material that offers numerous advantages for water purification. GO can effectively remove a broad range of pollutants, including organic dyes, aniline, heavy metals, and pharmaceutical compounds, due to the high adsorption capacity and the extensive surface area. Additionally, GO offers two important properties, i.e., regeneration ability and reusability,^18^ thereby reducing both treatment costs and environmental impact, suggesting GO as a sustainable solution for water treatment. The adjustable surface chemistry, substantial specific surface area, and capacity for both covalent and noncovalent functionalization position GO as a fundamental component of nanotechnology, bridging the gap between graphene’s exceptional intrinsic properties and its practical processability.^19^ Recent structural models, such as the Lerf–Klinowski model, emphasize the dynamic interplay between sp^3^-hybridized oxidized domains and sp^2^-conjugated regions within GO. This interaction plays a decisive role in determining the material’s electronic behavior, mechanical performance, and chemical reactivity.^20^ For this reason, these typical characteristics, such as tunable surface area and hybrid sp^3^–sp^2^ domains of GO, facilitate strong and selective interactions with organic pollutants, such as organic dyes, making them highly effective for their adsorption from wastewater.

Although the above properties of graphene constitute an important milestone, the scientific community increasingly utilizes graphene derivatives because of their enhanced surface chemistry and facile dispersion in various solvent media.^21^ One of the most comparable substitutes for graphene, i.e., GO or rGO, is attracting increasing attention.^22^ However, an important disadvantage derives from the high cost and limited scalability of GO material production as well as choosing synthesis methods that combine cost-effectiveness with chemical versatility.^23^ Hence, in response to these issues, there is a growing emphasis in current research on sustainable green synthesis approaches. Simultaneously, the synthesis of CS/MT/GO demonstrates significant potential for industrial applications in wastewater treatment due to its high adsorption capacity and the material’s regeneration capability.

In the literature, numerous adsorbent materials have been synthesized for the removal of organic dyes, such as CS/pencil clay (CCHB)^24^ for RB5 removal and charred parthenium (CP), a low-cost biomass-based adsorbent, for MB removal. However, although these adsorbent materials exhibit high removal efficiency, their adsorption capacity remains below 200 mg/g. The main objective of this research is to combine three nontoxic and low-cost adsorbent materials to develop a new composite with a high adsorption capacity. Specifically, increasing the adsorption capacity of a novel adsorbent material offers significant advantages, as it enables a high removal efficiency using a minimal amount of adsorbent, thereby reducing material costs. Furthermore, the scope of the present research is to develop a new composite material capable of maintaining high adsorption capacity even at high initial dye concentrations while being effective simultaneously for both anionic and cationic dyes, thus rendering the composite material more suitable for environmental and industrial applications.

This study synthesized the CS, MT, and GO blends to effectively remove RB5 and MB from aqueous (single-component and binary) solutions and real wastewater samples supplied from the textile industry. The synergic effect of the three materials (CS, MT, and GO) can increase the adsorption capacity due to their various functional groups and the extended surface area. Notably, CS offers biocompatibility and positive amino groups, and MT increases the ionic exchange,^25^ while GO contributes to strong intermolecular interactions, hydrogen bonds, or π–π* stacking interactions. The combination of CS, MT, and GO renders the CS/MT/GO composite an effective adsorbent for dye removal. According to the literature, Minisy et al. synthesized a CS–MT/polyaniline adsorbent to remove MB, achieving an adsorption capacity of 111.1 mg/g. In the present study, the combination of CS, MT, and GO targeted in a significantly higher adsorption capacity for dye removal.^26^ Overall, the proposed system represents a significant innovation in sustainable wastewater management, combining high efficiency, low cost, and environmental friendliness.

## Materials and Methods

### Materials

CS (high molecular weight, high deacetylation degree up to 75%, and purity up to 75%) was purchased from Sigma-Aldrich (Merck KGaA, Darmstadt, Germany). MT and sodium tripolyphosphate (TPP, purity up to 98%) were purchased from Thermo Scientific (Waltham, MA, U.S.A.). GO was synthesized in the lab using the Marcano method.^27^ With regard to the pollutants, RB5 (purities up to 50%) and MB (purities up to 82%) (Figure 1) were supplied by Sigma-Aldrich (Merck KGaA, Darmstadt, Germany). All the mentioned materials were in powder form. Finally, real textile wastewater was provided from a local textile dyeing industry (Thessaloniki, Greece).

**Figure 1:**
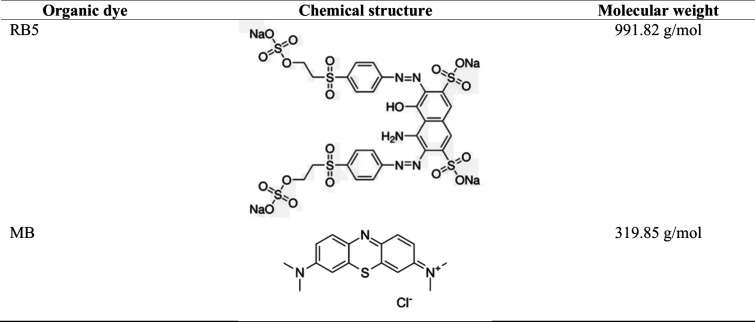
Chemical structures and molecular weights of RB5 and MB.

### Synthesis Process of Composites

The composites were synthesized by using a proposed green one-pot synthesis. Green synthesis is a modern approach to chemical synthesis that emphasizes sustainability, the reduction of toxic waste, and the use of environmentally friendly methods and materials. In addition, the one-pot method enables complex reactions to be completed in a single step, using simple raw materials without the need for intermediate separation.^28^ It is widely used in producing organic compounds due to its high efficiency, low cost, and ease of application.

#### Synthesis of CS/MT

A 1.5 g portion of MT was dispersed in a beaker with 300 mL of deionized water using a magnetic stirrer for 30 min (1000 rpm, 323 K). Then, 3 g of CS powder was added to the solution. When the CS powder was fully dispersed in an aqueous solution, 6 mL of acetic acid (2%, w/v) was added. Therefore, the mixture was left for 2 h under the same conditions (*T* = 333 K and 500 rpm) to ensure complete dissolution. Once the mixing process is complete and the formulation stability is confirmed, the solution is ready for the next step, which involves the formation of beads. During the experimental procedure, CS/MT ratios of 1:1, 1:2, and 2:1 were investigated. The 1:1 ratio was unstable in aqueous solution, and the beads rapidly disintegrated. The 2:1 ratio exhibited a higher adsorption capacity compared to the 1:2 ratio. For this reason, the incorporation of GO was performed using the optimal CS/MT ratio of 2:1.

#### Synthesis of CS/MT/GO

A total of 30 mg of GO was dissolved in 10 mL of double-distilled water using a sonicator for 1 h, and the GO solution was added to the mixture before the addition of CS in explanation. Specifically, the final mass ratio of CS/MT was 1:2, while the amount of GO corresponded to 1% of the CS mass. For the synthesis of GO using a modified version of Marcano’s method,^27^ initially, the oxidation of graphite by potassium permanganate (KMnO_4_) in the presence of a 9:1 mixture of sulfuric acid (H_2_SO_4_) and phosphoric acid (H_3_PO_4_) occurs.

#### Bead Formation

The final step of this experimental procedure involves the formation of the synthesized beads, as illustrated in Figure 2. The respective mixtures of new composites, CS/MT and CS/MT/GO, are removed from the magnetic stirring and heating plate and allowed to cool to room temperature.

**Figure 2:**
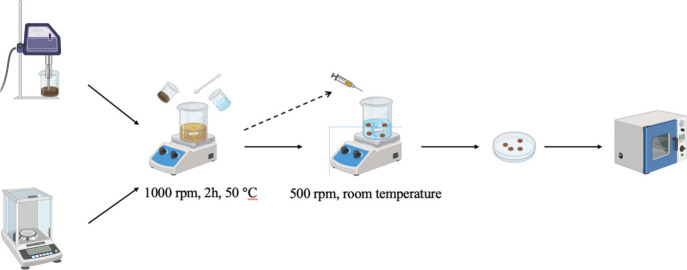
Synthetic processes of CS/MT and CS/MT/GO.

Subsequently, the mixtures are added dropwise into a separate beaker containing the cross-linker solution using a syringe. A TPP solution at 2% (w/v) was chosen as a cross-linker because it is nontoxic and forms ionically cross-linked networks with CS via electrostatic interactions.^29–31^ The TPP solution is stirred at room temperature during the addition of the composites. After the process, the beads are washed with double-distilled water to remove excess unreacted TPP. Finally, the beads are placed on a glass surface with filter paper and left to dry in an oven at 40 °C for a few hours, after which the adsorbent-dried beads are ready for adsorption evaluation.

### Characterization Techniques

The structural and physicochemical characterization of CS/MT and CS/MT/GO beads was carried out using Fourier transform infrared spectroscopy (FTIR), scanning electron microscopy (SEM), X-ray diffraction (XRD), and Brunauer–Emmett–Teller (BET) techniques. FTIR was performed to confirm the new chemical structure using a PerkinElmer Frontier ATR–FTIR (PerkinElmer, Shelton, CT, U.S.A.) spectrometer at a spectral range of 4000–550 cm^–1^. SEM characterization was performed using a JEOL JSM6390LV (Tokyo, Japan) scanning electron microscope to observe the beads’ surface morphology. XRD analysis was carried out using a Bruker D8 FOCUS X-ray diffractometer (Karlsruhe, Germany) with a 2θ range of 5–90° to study the crystallinity of the new composites. Lastly, the BET technique was performed using a NOVA 4200a (Boynton Beach, FL, U.S.A.) analyzer to study the general porosity of the beads.

### Adsorption Evaluation

#### Effect of pH

Studying the effect of pH is a fundamental aspect of all batch experiments, because pH determines all other parameters related to adsorption. Thus, 20 mg of CS/MT and CS/MT/GO beads was added to a 20 mL conical flask containing an aqueous dye solution and tested at a 2.0–12.0 pH range. According to the literature, the most common initial concentration of RB5 and MB is 250 mg/L^49^ and 100 mg/L,^32^ respectively. The conical flasks were placed in a water bath at 303 K and stirred at 150 rpm for 24 h. The effectiveness of the adsorbents was evaluated by assessing both the removal efficiency and the adsorption capacity under different experimental conditions.

The removal percentage (*R*, %) of RB5 and MB was calculated as shown in eq 1

$$R=\frac{\left(C_{0}-C_{e}\right)}{C_{0}}\times 100\%$$ 1

where *R* is the removal percentage (%) and *C*_0_ and *C*_e_ are the initial and equilibrium concentrations of the pollutant (mg/L).

Τhe amount of pollutant adsorbed by CS/MT and CS/MT/GO is given in eq 2

$$Q_{e}=\frac{\left(C_{0}-C_{e}\right)V}{m}$$ 2

where *Q*_e_ is the adsorption capacity (mg/g), *C*_0_ is the initial concentration of the pollutant (mg/L), *C*_e_ is the concentration of the pollutant at a dedicated time, *V* is the volume of the solution (L), and *m* (g) is the mass of the adsorbent.

The experiments were repeated 3 times to ensure that the results were consistent and reliable, indicating that the observed effects were not due to random chance or experimental error.

#### Kinetic Study

Continuing the evaluation of adsorption, a kinetic study was carried out to determine the behavior of the adsorbents. Two kinetic adsorption models, pseudo-first order (PFO) and pseudo-second order (PSO), are used, as shown in eqs 3 and 4. Among them, physical adsorption, i.e., the weak intermolecular contact between the pollutant and the adsorbent, is specifically described by the PFO kinetic model.^33^ On the other hand, chemisorption appears to be the creation of a new ionic or covalent bond between the pollutant and the adsorbent material, which is explained by the PSO kinetic model.^34^ The correlation coefficient (*R* ^2^) was chosen as a critical criterion to determine which model fits the experimental data better

$$Q_{t}=Q_{e}\left(1-e^{-K_{1}t}\right)$$ 3

$$Q_{t}=\frac{{Q_{e}}^{2}K_{2}t}{\left(1+Q_{e}K_{2}t\right)}$$ 4

where the first- and second-order rate constants are *K*_1_ (min^–1^) and *K*_2_ (g mg^–1^ min^–1^), respectively, and *Q*_e_ and *Q*_*t*_ (mg/g) represent the adsorption at equilibrium and time *t*.

During the kinetic experiments, the ratio between the adsorbent and the pollutant remained at 1 g/L and the samples were tested at regular intervals of 5, 10, 15, 20, 30, 45, 60, 90, 120, 180, 360, 720, and 1440 min. Moreover, for the adsorption process in aqueous solution, the initial concentrations of RB5 and MB were 250 and 100 mg/L, respectively, and the temperature was 303 K. However, the two dyes were added in equal molar amounts in the dye mixture solution, maintaining a 1:1 ratio.

#### Isotherm Study

Isotherm models provide a quantitative description of the adsorption process, elucidating the relationship between the adsorbate concentration and adsorption capacity at equilibrium. In recent decades, equilibrium models have been developed to describe the relationship between isotherms under the equilibrium conditions. This study describes the most commonly applied Langmuir^46^ and Freundlich^36^ models. In detail, the Langmuir model (eq 5) is appropriate for monolayer adsorption on homogeneous sites, while the Freundlich model (eq 6) is referred to as multilayer adsorption on heterogeneous sites

$$Q_{e}=\frac{K_{L}Q_{m}C_{e}}{\left(1+K_{L}C_{e}\right)}$$ 5

$$Q_{e}=K_{F}{Ce}^{1/n}$$ 6

where *Q*_e_ is the solute mass adsorbed per unit of adsorbent mass at equilibrium (mg/g), *Q*_m_ represents the maximum adsorption capacity (mg/g), *C*_e_ is the concentration of the pollutant at equilibrium (mg/L), *K*_L_ the constant of the Langmuir isotherm (L/mg), *K*_F_ is the constant of the Freundlich isotherm (L^1/*n*^ mg^(1–1)/*n*^ g^–1^), and 1/*n* is the adsorption intensity.

During the isotherm study, the adsorbent amount was 1 g/L, and the experiments were carried out under the optimum conditions. The different initial concentrations of RB5 and MB were 50–1250 and 25–250 mg/L, respectively.

#### Thermodynamic Analysis

Thermodynamic studies were conducted to evaluate the adsorption mechanism further. Understanding thermodynamic assessments is essential to determine the nature of the process, specifically whether it is spontaneous. The change in Gibbs free energy is described by eqs 7 and 8 ^37^

$${\Delta G}^{0}=-RT\ln\left(K_{c}\right)$$ 7

$${\Delta G}^{0}={\Delta H}^{0}-{T\Delta S}^{0}$$ 8

where *R* is the gas constant (8.314 J mol^–1^ K^–1^), *T* represents the temperature (K), and *K*_c_ is the thermodynamic constant.

The experiments were repeated at three different temperatures (303, 313, and 323 K), and thermodynamic parameters (Δ*H*° and Δ*S*°) were determined from eqs 9 and 10.^38^

$$K_{c}=\frac{C_{s}}{C_{e}}$$ 9

$$\ln\left(K_{c}\right)=\left(-\frac{{\Delta H}^{{}^{\circ}}}{RT}\right)+\frac{{\Delta S}^{{}^{\circ}}}{R}$$ 10

## Results and Discussion

### Characterization Analysis

#### Fourier Transform Infrared Spectroscopy (FTIR)

FTIR spectra were used to identify the successful synthesis of the composite beads CS/MT and CS/MT/GO, as shown in Figure 3. The spectrum of CS (Figure 3a) exhibited distinct bands, such as a broad range at 3200–3500 cm^–1^ corresponding to O–H and N–H vibrations and a band near 2930 cm^–1^ attributed to aliphatic C–H stretching.^39^ Moreover, a band at 1022 cm^–1^ is associated with C–O–C stretching, and another at 1651 cm^–1^ represents N–H bending vibrations of the NH_2_ group.^39^ Figure 3b presents the characteristic peaks of MT, including stretching bands of structural hydroxyl groups at 3640 cm^–1^, a Si–O stretching band at 1040 cm^–1^, Al–Al–OH deformation bands at 915 cm^–1^, an Al–Mg–OH deformation band at 840 cm^–1^, and a silica Si–O stretching band at 625 cm^–1.^ ^40^

**Figure 3:**
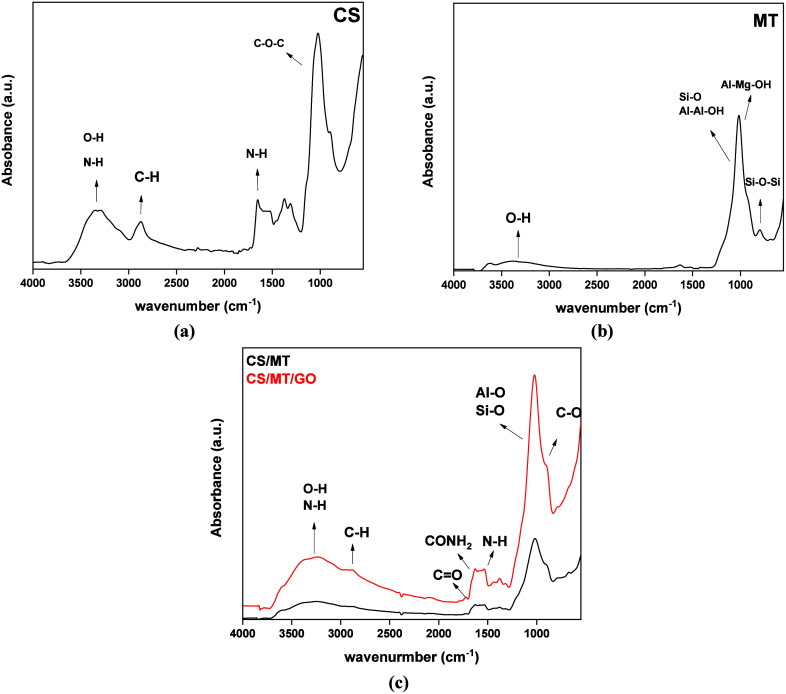
FTIR spectra of (a) CS, (b) MT, and (c) CS/MT and CS/MT/GO beads.

The FTIR spectrum of the CS/MT composite, shown in Figure 3c, retains all of the essential features of both components, with no new peaks observed. In particular, the C–H bond vibration appeared at 2930 cm^–1^ in the CS spectrum and shifted to 2886 and 2868 cm^–1^ in the CS/MT and CS/MT/GO spectra, respectively.^41^ Additionally, the peak shift from 1651 cm^–1^ to 1634 and 1621 cm^–1^ corresponds to the vibration of amino groups in the CS, CS/MT, and CS/MT/GO spectra, respectively. The slight displacement of the vibrational peak is likely due to weak intermolecular van der Waals or electrostatic interactions developed between CS.^42^

Furthermore, the increased intensity in CS/MT/GO is due to the hydroxyl groups of GO in the composite material.^43^ The presence of MT could be identified by the peaks from 1000 to 500 cm^–1^, representing stretching vibrations of Al–O and Si–O groups and bending vibrations of Al–O–Si and Si–O–Si, respectively.^40^ The peak at 1710 cm^–1^ appears in the CS/MT/GO spectrum and indicates the vibration of the C=O bond. This covalent chemical bond, C=O, arises from the interaction between the functional groups of GO and the hydroxyl and amino groups of CS.^44^

In comparison of the FTIR spectra before and after adsorption of RB5 in Figure 4a and b, a peak shift from 1529 to 1493 and 1488 cm^–1^ was observed for composite materials CS/MT and CS/MT/GO, respectively. In Figure 4c, a similar peak shift from 1560 to 1530 cm^–1^ was noticed in the FTIR spectra after MB adsorption, while Figure 4d shows a peak alteration from 1542 to 1529 cm^–1^. This displacement of vibrational peaks is attributed to the hydrogen bond between adsorbents and organic dyes.^45^ In general, the peaks before and after adsorption show significant changes, which suggests that the phenomenon primarily involves hydrogen bonds and π–π* stacking between the beads and the dyes.

**Figure 4:**
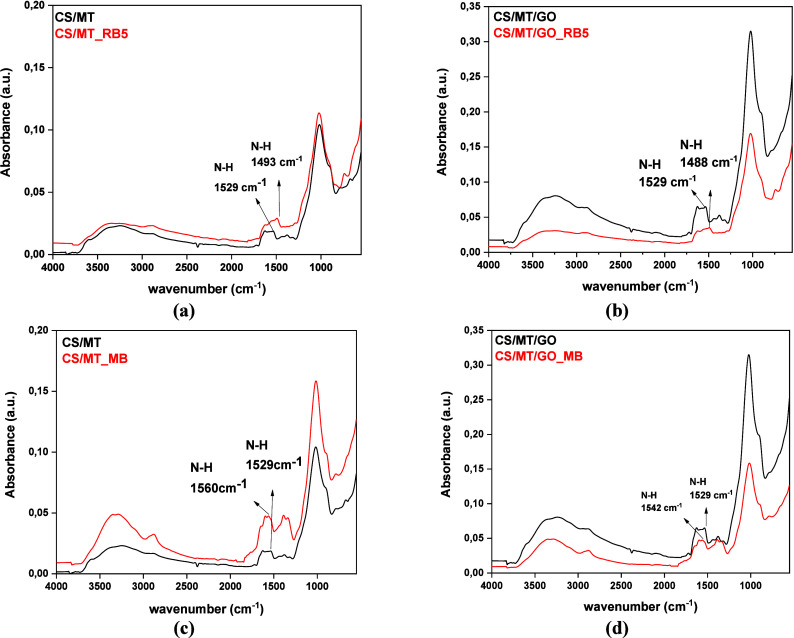
FTIR spectra of CS/MT and CS/MT/GO before and after adsorption of (a and b) RB5 and (c and d) MB, respectively.

#### X-ray Diffraction (XRD)

The crystallinity of the structure was analyzed using the XRD characterization method. Figure 5 presents the relative diagrams of CS/MT and CS/MT/GO. As observed, the addition of GO affects the crystallinity of the composite, because GO has a more regular and organized structure. This is evident from the higher intensity and sharper peaks observed in the CS/MT/GO pattern compared to the CS/MT spectrum. According to the literature, the peak at 2θ of 20° corresponds to CS and is mainly associated with intermolecular interactions between polymeric chains.^46^ In addition, characteristic peaks of MT are observed at 2θ of 5.3°, 26°, and 35°, which are of very low intensity, indicating the low crystallinity of the adsorbent material.^47^ The addition of GO to the polymeric matrix causes changes in the degree and appearance of new peaks.^48^ In this case, these peaks appear at lower degrees in the CS/MT/GO diagram, indicating that GO has penetrated the interior of the structure. Finally, the 2θ of 10° peak^49^ is present only in the CS/MT/GO diagram and demonstrates the presence of GO. It is concluded that the addition of GO positively affected the composite structure.

**Figure 5:**
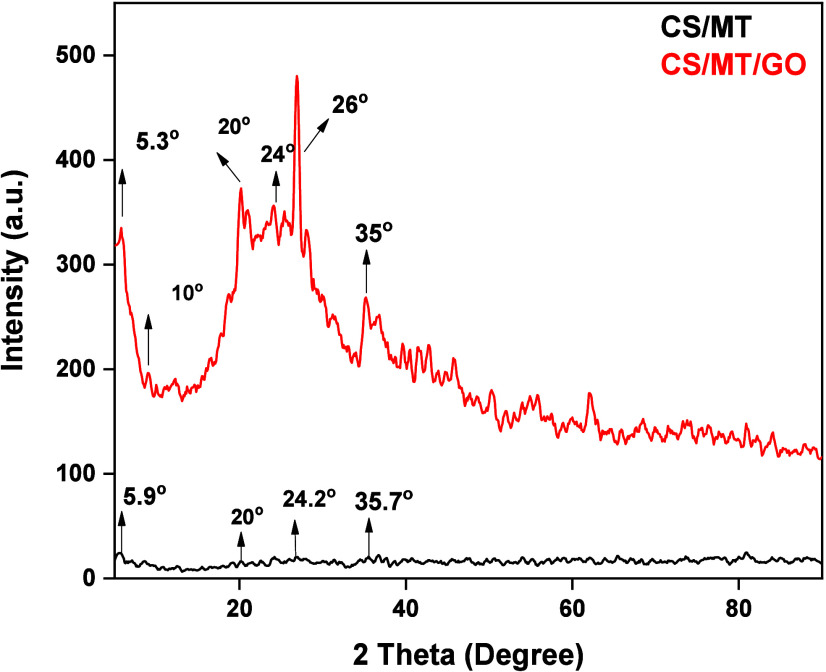
XRD diagrams of CS/MT and CS/MT/GO.

#### Scanning Electron Microscopy (SEM)

The surface of the composite beads was observed and characterized by SEM. All the images were taken with a voltage of 15 kV, as shown in Figure 6.

**Figure 6:**
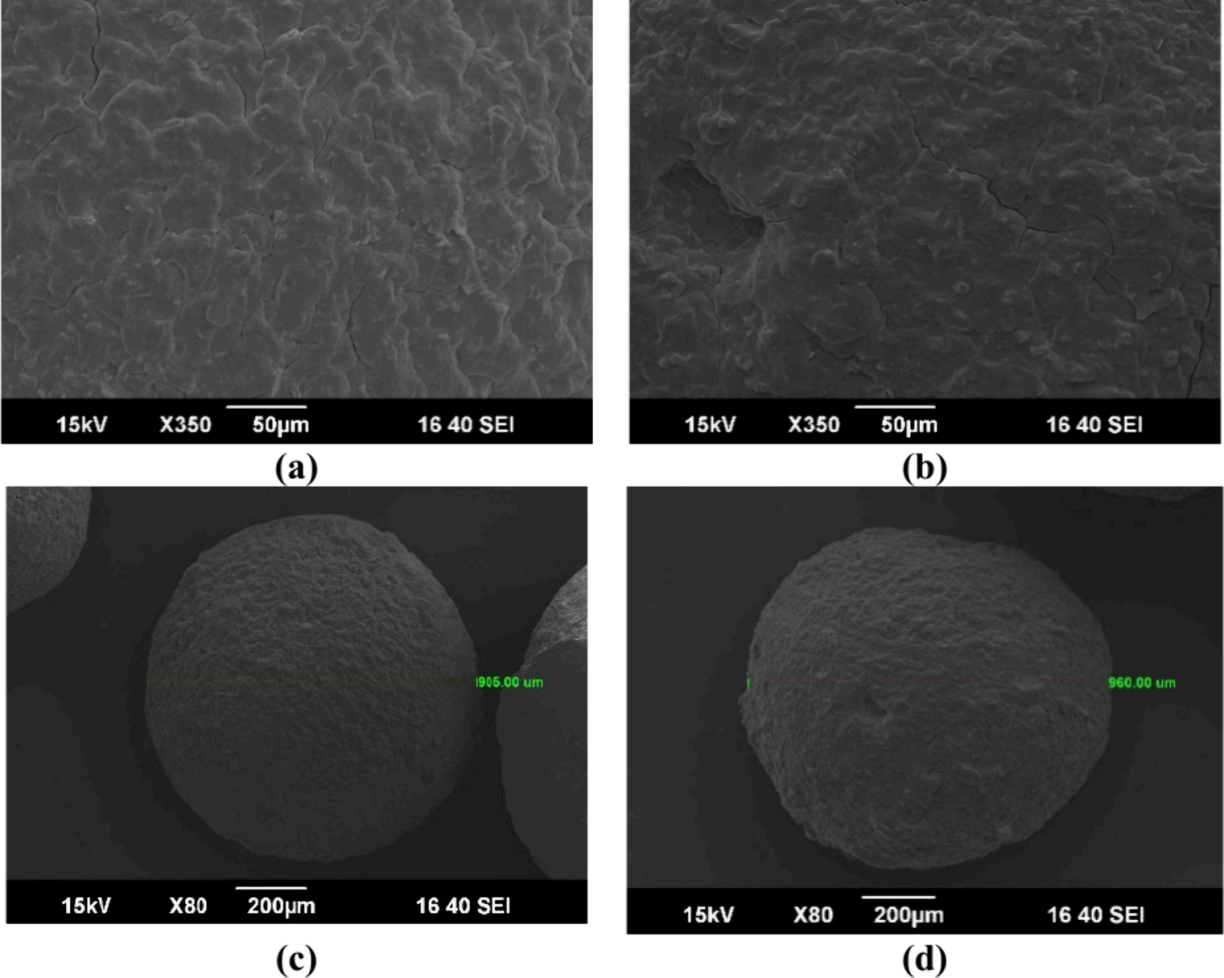
SEM images of (a) CS/MT, (b) CS/MT/GO, (c) CS/MT beads, and (d) CS/MT/GO beads.

Therefore, Figure 6a and b presents the SEM images of CS/MT and CS/MT/GO. The morphology of CS/MT/GO appears smoother than that of CS/MT. This phenomenon suggests that GO was homogeneously distributed within the CS/MT polymeric matrix.^50^ In addition, structural modifications within the hydrogel matrix during the freeze-drying process may cause cracks on the surface.

Figure 6c and d presents the diameters of the CS/MT and CS/MT/GO beads. In this case, the diameters of the dried CS/MT and CS/MT/GO beads are 0.90 and 0.96 mm, respectively. As observed, the surface of the beads is uniformly rough, which contributes to an increased surface area and, as confirmed by BET, improves the adsorption efficiency of the material.

#### Brunauer–Emmett–Teller (BET)

BET analysis was carried out using nitrogen adsorption–desorption isotherms to investigate the specific surface area of the beads, their pore size, and their distribution. More specifically, as shown in Figure 7, according to the IUPAC standard isotherms, both CS/MT and CS/MT/GO beads exhibit a type III isotherm, indicating that the adsorbed molecules are concentrated around the most favorable sites on the surface of the macroporous beads.^51^

**Figure 7:**
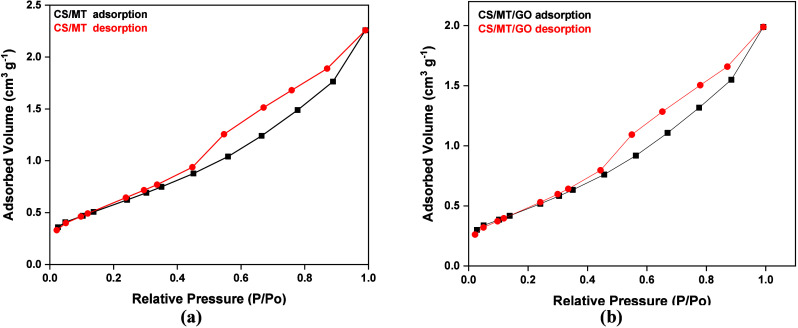
BET diagram of (a) CS/MT beads and (b) CS/MT/GO beads.

In addition, the most relevant hysteresis loop is H3, which is associated with clay materials, as is the case with much of the adsorbent material. Moreover, Table 1 shows a general increase in the composite beads compared to CS neat. Specifically, the surface area increases from 20.1 to 28.83 and 44.61 m^2^/g for CS/MT and CS/MT/GO, respectively, which accounts for the enhanced adsorption capacity. Finally, as reported in the literature, the average pore diameter increases from 14.77 to 40.56 and 40.59 nm for CS/MT and CS/MT/GO, respectively.

**Table 1:** BET Analysis of CS/MT Beads, CS/MT/GO Beads, and CS Neat

adsorbent	surface area (m^2^/g)	pore volume (cm^3^/g)	average pore diameter (nm)
CS/MT	28.83	0.035	40.56
CS/MT/GO	44.61	0.055	40.59
CS neat	20.10	0.076	14.77

#### Effect of pH

The initial solution pH is a significant factor that defines the adsorption process. Figure 8 shows the effect of the pH on the adsorption of RB5 and MB. RB5 is considered an anionic dye mainly because of the −SO_3_Na groups in its chain,^52^ which can easily be ionized under acidic conditions and interact with other positively charged polymers, such as CS. On the other hand, MB is a cationic dye and is mainly positively charged under alkaline conditions.^53^ MB can interact with CS through hydroxyl groups and hydrogen bonds and also through π–π* interactions.

**Figure 8:**
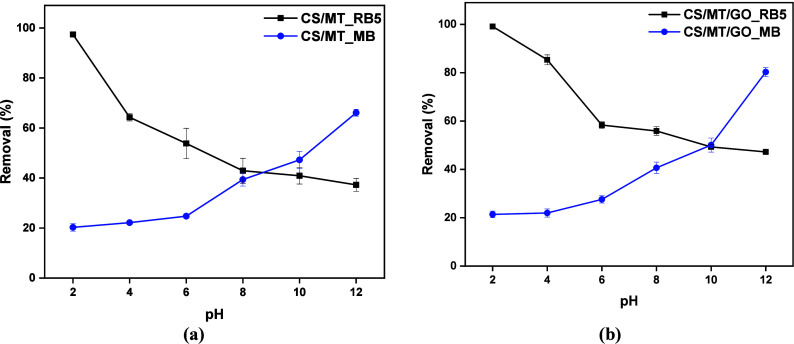
Effect of pH on the adsorption of RB5 (*C*_0_ = 250 mg/L, 1 g/L, 24 h, and 303 K) and MB (*C*_0_ = 100 mg/L, 1 g/L, 24 h, and 303 K) onto (a) CS/MT and (b) CS/MT/GO beads.

To investigate the effect of pH, the samples were tested over a pH range of 2–12. The results showed that the optimal initial pH for RB5 adsorption was 2, as the removal efficiencies reached 97.3% for CS/MT and 99.1% for CS/MT/GO. In addition, MB removal reached 66.18% for CS/MT and 80.29% for CS/MT/GO at pH 12. The optimal initial pH of this experiment meets the literature,^54^ as CS-based material was synthesized for both of these dyes and found the same optimum pH values.

Moreover, the point of zero charge (pH_pzc_) is determined according to the pH drift method,^55^ as shown in Figure 9. After the calculations, the pH_pzc_ values for CS/MT and CS/MT/GO beads were 7.05 and 6.99, respectively. pH_pzc_ is essential to describe the surface interaction between the adsorbent material and organic dyes. According to the literature, when pH > pH_pzc_, the adsorbent surface is negatively charged, while when pH < pH_pzc_, the adsorbent surface is positively charged.^56^ In this study, the optimum pH values for the adsorption process are 2 and 12 for RB5 and MB, respectively. Specifically, at pH value of 2, CS/MT/GO offers a positive surface area and interacts with anionic organic dye (RB5) via van der Waals forces, electrostatic interactions, and hydrogen bonding. On the other hand, at pH value of 12, CS/MT/GO presents a negative surface area and interacts with cationic MB dye through different intermolecular interactions (van der Waals forces, electrostatic interactions, etc.).^57^ In addition, graphene oxide has a crucial role in enhancing the overall performance of the composite by providing hydrophilic functional groups, such as hydroxyl (−OH), carboxyl (−COOH), and epoxy groups,^58^ while the hybrid sp^3^–sp^2^ domains of GO interact with extended aromatic systems of organic pollutants. These characteristic groups improve dispersion in aqueous solutions and generate active adsorption sites. Additionally, interactions, including van der Waals forces, hydrogen bonding, and π–π* stacking, with aromatic dye molecules contribute to the adsorption mechanism, as shown in Figure 10.

**Figure 9:**
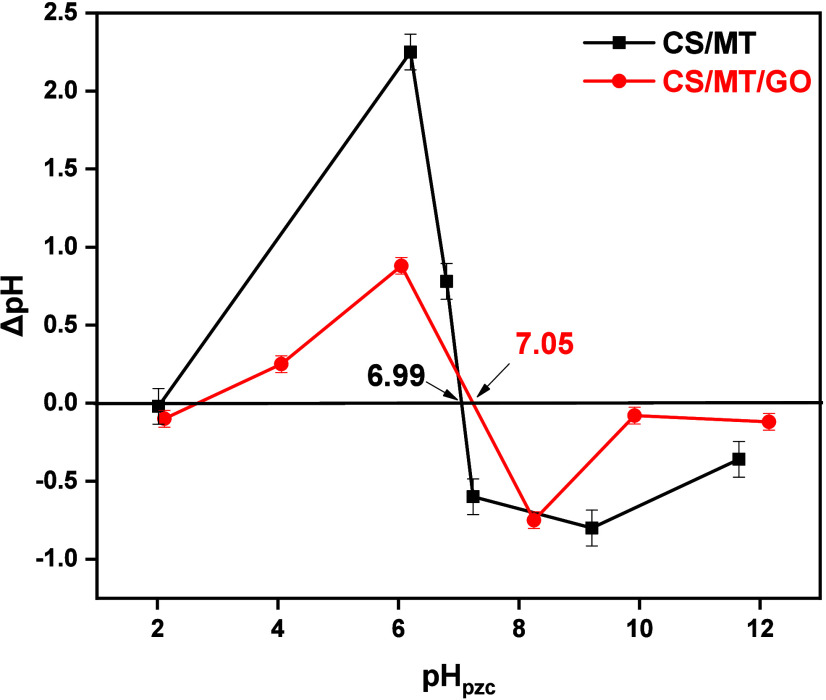
Determination of pH_pzc_ of CS/MT and CS/MT/GO using the pH drift method (NaNO_3_ = 0.1 M, pH values of 2.0, 4.0, 6.0, 8.0, 10.0, and 12.0, adsorbent dosage of 1 g/L, and 303 K).

**Figure 10:**
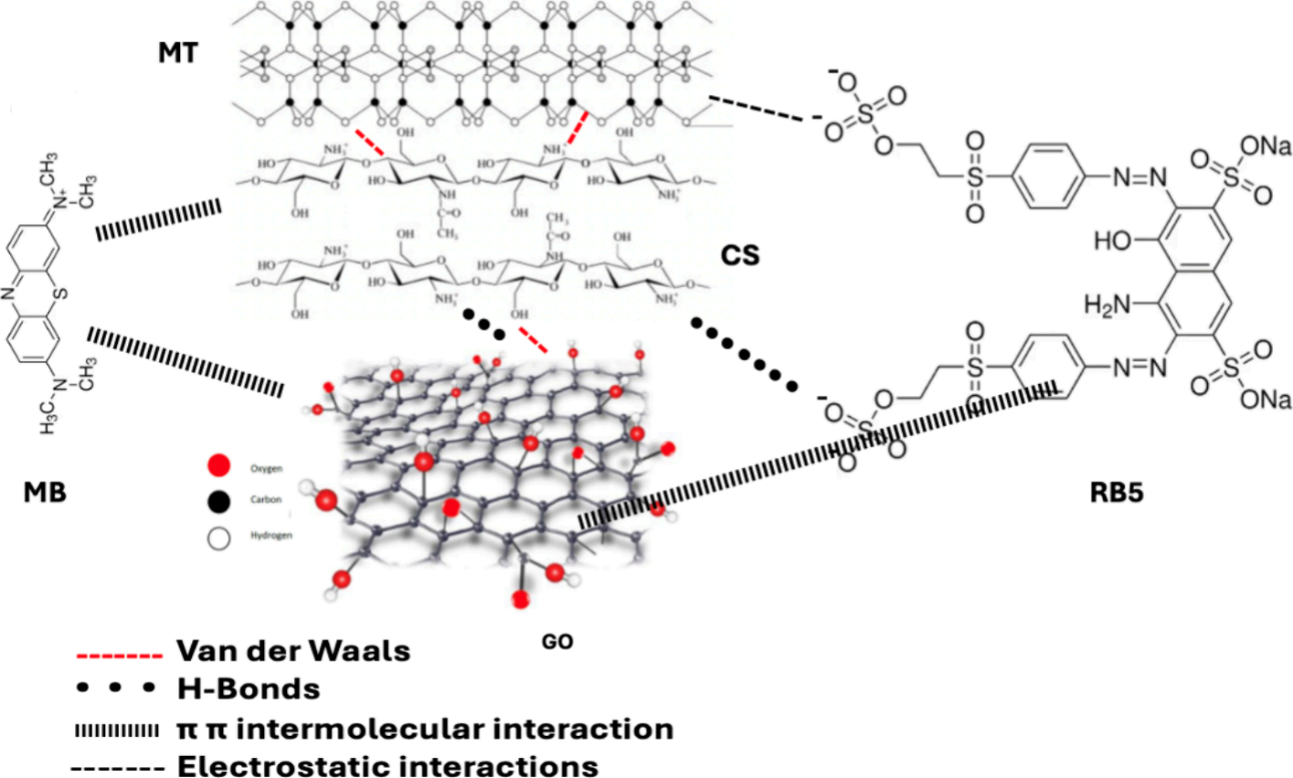
Proposed adsorption mechanism of the dyes RB5 and MB onto the CS/MT/GO composite material, along with their corresponding intermolecular interactions, including electrostatic forces, hydrogen bonding, and π–π stacking.

#### Effect of the Contact Time

The contact time is a critical parameter for evaluating the kinetics of adsorption because it determines the time required for the maximum amount of pollutant to be adsorbed. Figure 11 summarizes the effect of the contact time on RB5 and MB adsorption. Two common kinetic models, PFO and PSO, are used to understand the adsorption type.

**Figure 11:**
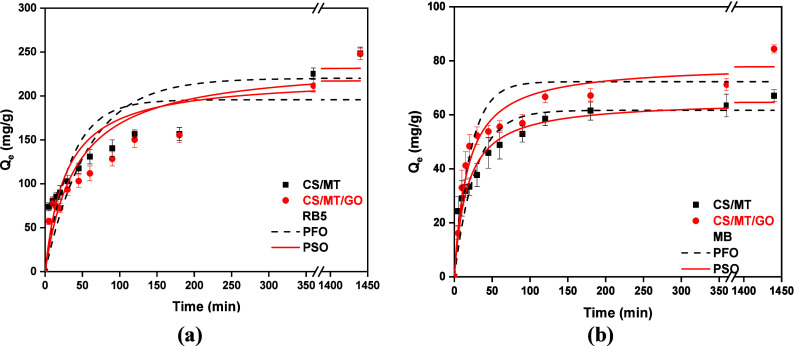
Kinetic study of (a) RB5 (*C*_0_ = 250 mg/L, pH 2, and *T* = 303 K) and (b) MB (*C*_0_ = 100 mg/L, pH 12, and *T* = 303 K).

As observed (Figure 11), 50% of dye is removed in the first 45 min for RB5 (Figure 11a) and MB (Figure 11b) during 60 min. After this point, the removal rate decreases for both dyes, with equilibrium reaching 300 min for RB5 and 180 min for MB. After the PFO and PSO experimental models were applied (Table 2), it was found that PSO, which had the highest correlation coefficient, fits the experimental data better. Therefore, the kinetic study of CS/MT and CS/MT/GO for RB5 and MB adsorption is described by chemisorption.

**Table 2:** PFO and PSO Kinetic Parameters for RB5 and MB Adsorption

		pseudo-first-order model			pseudo-second-order model		
dye	adsorbent	*k*_1_ (min^–1^)	*Q*_e_ (mg/g)	*R* ^2^	*k*_2_ (L mg^–1^ min^–1^)	*Q*_e_ (mg/g)	*R* ^2^
RB5	CS/MT	0.029	195.71	0.920	0.000164	221.27	0.960
	CS/MT/GO	0.017	222.11	0.933	0.000101	238.14	0.962
MB	CS/MT	0.036	61.68	0.977	0.000915	65.35	0.992
	CS/MT/GO	0.048	72.27	0.977	0.000737	78.72	0.991

#### Isotherm Study

Two widely used isotherm models were applied to describe the adsorption process effectively. The Freundlich isotherm model is one of the best known mathematical models for explaining how a molecule physically adsorbs onto a surface. It is commonly used to analyze heterogeneous adsorption on surfaces with different energy distributions. In contrast, the Langmuir model describes the monolayer adsorption of molecules on a homogeneous surface.^35^ The highest correlation efficiency (*R* ^2^) determines which isotherm model fits better in the experimental data. Another criterion that confirms that adsorption best fits the Freundlich model is the SSE method.^59^ According to this method, a lower SSE value indicates a better correlation with the adsorption model. More specifically, the adsorption of dyes RB5 and MB onto the CS/MT/GO composite is better described by the Freundlich model, as indicated by the higher *R* ^2^ (0.979 for RB5 and 0.986 for MB) and lower SSE values (138763 for RB5 and 26839 for MB). On the other hand, the corresponding *R* ^2^ (0.974 for RB5 and 0.958 for MB) and SSE values for the Langmuir model are higher (152246 for RB5 and 27723 for MB), indicating a less accurate representation of the experimental data. As shown in Figure 12 and Tables 3 and 4, the Freundlich isotherm model fits better than Langmuir on experimental data for RB5 (Figure 12a) and MB (Figure 12b) adsorption. Τhe maximum adsorption capacities (*Q*_m_) were calculated to be 317.96 mg/g for RB5 and 203.98 mg/g for MB, respectively, with the optimum adsorbent material CS/MT/GO.

**Figure 12:**
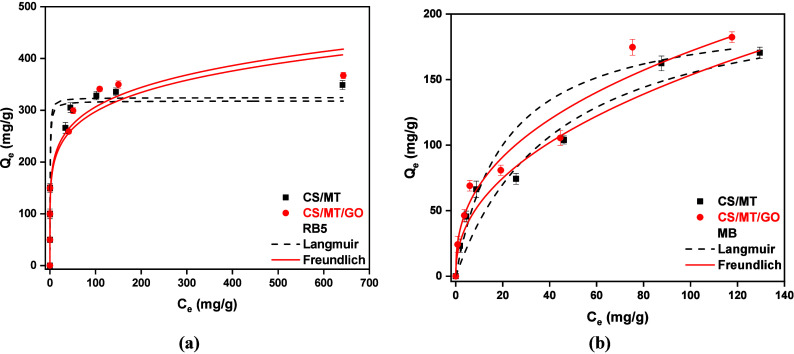
Isotherm study of (a) RB5 (*C*_0_ = 50–800 mg/L, pH 2, *t* = 300 min, and adsorbent dosage of 1 g/L) and (b) MB (*C*_0_ = 50–150 mg/L, pH 12, *t* = 180 min, and adsorbent dosage of 1 g/L).

**Table 3:** Langmuir Isotherm Model Parameters for CS/MT and CS/MT/GO Adsorption Materials

Langmuir model				
pollutant	adsorbent	*Κ*_L_ (L/mg)	*R* ^2^	*Q*_m_ (mg/g)
RB5	CS/MT	2.10	0.989	324.40
	CS/MT/GO	1.60	0.974	317.96
MB	CS/MT	0.0230	0.968	222.35
	CS/MT/GO	0.0481	0.958	203.98

**Table 4:** Freundlich Isotherm Model Parameters for the CS/MT and CS/MT/GO Adsorption Materials

Freundlich model				
pollutant	adsorbent	*Κ*_F_ (mg/g) (L/mg)^1/*n*^	*R* ^2^	1/*n*
RB5	CS/MT	136.65	0.968	0.168
	CS/MT/GO	142.10	0.979	0.166
MB	CS/MT	19.56	0.990	0.446
	CS/MT/GO	27.87	0.986	0.395

The Freundlich constant (1/*n*) represents the adsorption intensity and is categorized as follows: very easy to adsorb (0 < 1/*n* ≤ 0.5), easy to adsorb (0.5 < 1/*n* ≤ 1), and challenging to adsorb (1/*n* > 1). Table 4 shows that the adsorption process is a possible interaction between the adsorbent materials and pollutants. More specifically, the 1/*n* value reveals that the anionic dye RB5 is adsorbed more easily onto the composites CS/MT and CS/MT/GO compared to the cationic dye MB. According to the Freundlich parameter, CS/MT/GO strongly adsorbs the anionic dye (1/*n* = 0.166) rather than the cationic dye (1/*n* = 0.395). This phenomenon is likely attributed to the intermolecular interactions between the positively charged surface of CS/MT/GO and the negatively charged RB5 molecules, as suggested in the proposed adsorption mechanism described previously.

#### Thermodynamic Study

Several thermodynamic parameters were investigated during the adsorption process. Experiments were conducted at 303, 313, and 323 K. The negative Δ*G* ^0^ values observed for the CS/MT and CS/MT/GO composites with RB5 and MB dyes indicate that the adsorption process proceeds spontaneously under the studied conditions.^60^

With the observation of the values of Δ*H* ^0^ and Δ*S* ^0^ (Table 5), it can be concluded that the adsorption of RB5 and MB is mostly endothermic and the process is not accompanied by a significant change in the level of randomness at the solid–liquid interface.^61^ Finally, the ln *K*_c_ values obtained indicate a high thermodynamic preference of the adsorbent for the removal of RB5 and MB dyes. In particular, the highest value of 5.64, which was presented during RB5 adsorption onto CS/MT/GO, indicates a stronger adsorption for the corresponding pollutant, implying increased exchanges between the adsorbent and the dye. The alteration in ln *K*_c_ values for the pollutants (5.64 and 0.82 for RB5 and MB at 313 K, respectively) can be attributed to the distinct intermolecular interactions that occur between the anionic and cationic organic dyes and the adsorbent material CS/MT/GO.

**Table 5:** Thermodynamic Parameters for the Adsorption of RB5 and MB onto CS/MT/GO Beads

pollutant	*T* (K)	Δ*G* ^0^ (kJ/mol)	Δ*H* ^0^ (kJ/mol)	Δ*S* ^0^ (kJ mol^–1^ K^–1^)	ln *K*_c_
RB5	303	–11.78	20.97	0.110	4.67
	313	–14.69			5.64
	323	–13.90			5.17
MB	303	–3.62	20.24	0.018	1.44
	313	–4.13			0.82
	323	–5.25			1.95

#### Reuse of CS/MT/GO Beads

Regeneration of adsorbent materials is significant for environmental sustainability because it allows water reuse in cleaning processes and reduces costs and waste generation. Figure 13 presents the regeneration results of CS/MT/GO for RB5 (Figure 13a) and MB (Figure 13b) after 5 regeneration cycles.

**Figure 13:**
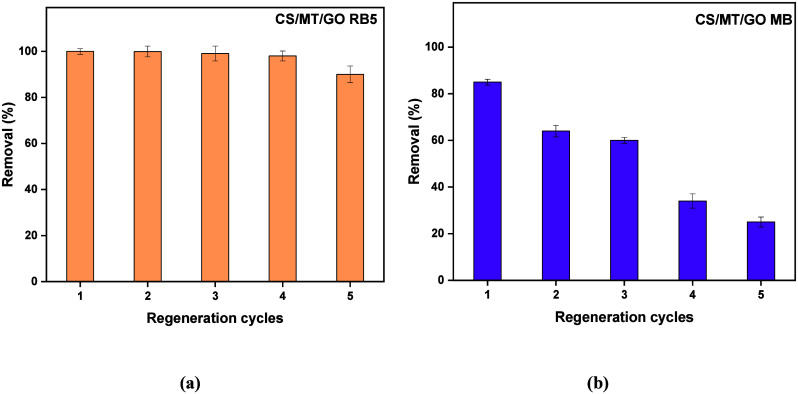
Regeneration of (a) RB5 and (b) MB from adsorbent material CS/MT and CS/MT/GO beads (for RB5, pH_ads_ 2, pH_des_ 12, time = equilibrium, *C*_0_ = 250 mg/L, and *T* = 303 K; for MB, pH_ads_ 12, pH_des_ 4, time = equilibrium, *C*_0_ = 100 mg/L, and *T* = 303 K).

The reusability of CS/MT/GO for removing RB5 and MB was tested through adsorption–desorption experiments. In the regeneration process, a 0.01 M NaOH solution is used for dye removal from adsorbent beads. According to the literature, regeneration experiments have demonstrated that these alkaline conditions are effective for the adsorption and desorption processes.^62^ In the first cycle, CS/MT/GO removed 99% of RB5 and 80% of MB. However, after 5 regeneration cycles, besides the fact that CS/MT/GO still removed 90% of RB5, the recovery capability for MB has decreased to 20%. Due to its positive charge surface area, CS/MT/GO is more effective in removing RB5 due to heteropolar intermolecular interactions between the adsorbent and the pollutant. Therefore, CS/MT/GO is an effective, regenerable, and cost-effective adsorbent material for wastewater treatment, capable of removing anionic organic dyes without losing its high adsorption performance over multiple uses.

### Adsorption in Binary Dyeing Systems

The adsorption process of CS/MT and CS/MT/GO was investigated in a mixture solution of RB5 and MB in a 1:1 molar ratio (for comparison purposes). According to the literature, a synergetic effect occurs when the presence of one dye enhances the adsorption of another. At the same time, the competitive impact arises when organic dyes compete for the same active sites on the adsorbent surface.^63^ As shown in Figure 14, MB adsorption on CS/MT and CS/MT/GO increased (from 66.1 to 90.2% and from 80.3 to 90.6%, respectively) in the presence of RB5 (synergistic effect). On the other hand, RB5 adsorption was reduced (from 97.4 to 96.6% and from 99.1 to 96.5%) due to the presence of MB. Therefore, in the mixture solution, the presence of an anionic dye positively influences the removal of MB, whereas the opposite phenomenon is observed in the case of RB5. A similar phenomenon is present in the literature during organic dye adsorption from natural adsorbent materials (banana, orange, and pomegranate).^64^

**Figure 14:**
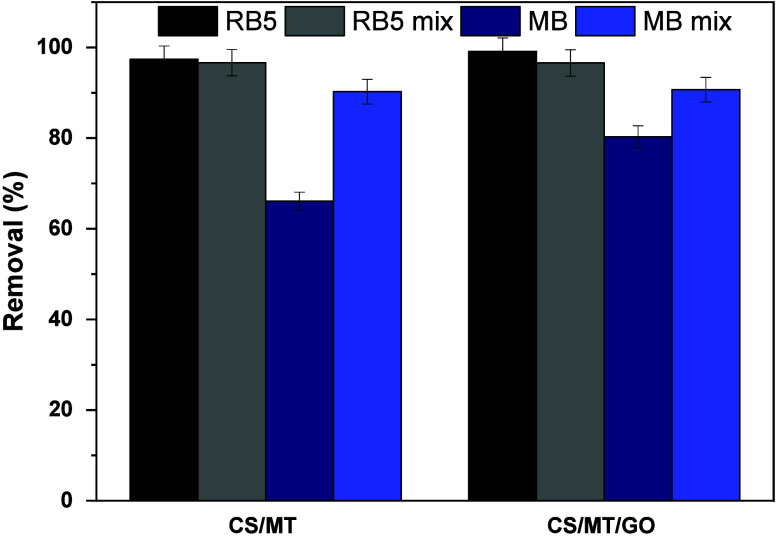
Evaluation of CS/MT and CS/MT/GO for the removal of RB5 and MB individually and in mixed dye solutions (pH free, dose of 1 g/L, *T* = 303 K, and contact time of 24 h).

#### Kinetic Study

A kinetic study of RB5 and MB in the dyeing mixtures was investigated. The experimental conditions applied were a contact time of 5–1440 min, adsorbent dosage of 1 g/L, pH value free, and 303 K. Figure 15 shows the effect of the contact time in the adsorption of mixture solutions from CS/MT and CS/MT/GO, while Table 6 presents the kinetic parameters for the adsorption process in the binary system. In this experiment, the equilibrium time (Figure 15) for the dye mixture is longer than 120 min. In contrast, for the individual RB5 and MB solutions, it is 45 min (Figure 12a) and 60 min (Figure 12b), respectively. During adsorption from a binary system, competitive interactions may arise between different adsorbates to access the limited number of available adsorption sites. The presence of one organic pollutant can significantly hinder the adsorption efficiency of other coexisting dyes, impeding the adsorption efficiency of other dyes. In the present case, MB (Figure 15b) exhibited higher adsorption efficiency than RB5 (Figure 15a) due to its lower molecular weight and simple chemical structure, which may facilitate greater interaction with the adsorbent surface. Finally, the CS/MT/GO composite demonstrated superior removal efficiency of organic pollutants, attributed to its enhanced specific surface area compared to CS/MT.

**Figure 15:**
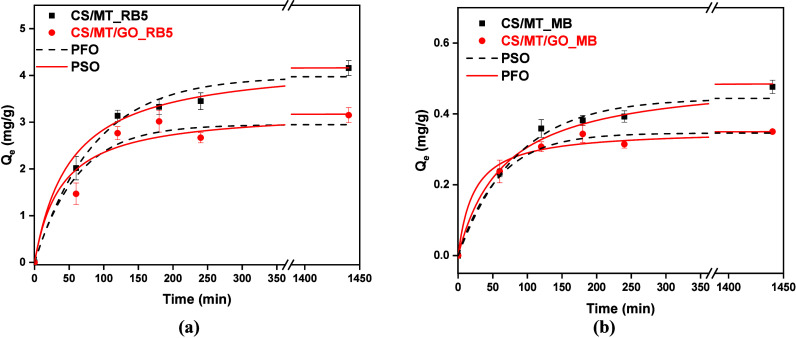
Adsorption process regarding (a) RB5 and (b) MB as a binary system.

**Table 6:** Kinetic Parameters for the Adsorption Process in a Binary System

		pseudo-first-order model			pseudo-second-order model		
mix dye	adsorbent	*k*_1_ (min^–1^)	*Q*_e_ (mg/g)	*R* ^2^	*k*_2_ (L mg^–1^ min^–1^)	*Q*_e_ (mg/g)	*R* ^2^
RB5	CS/MT	0.011	3.97	0.887	0.00446	4.31	0.995
	CS/MT/GO	0.016	2.94	0.673	0.00797	3.25	0.710
MB	CS/MT	0.011	0.443	0.994	0.030	0.51	0.977
	CS/MT/GO	0.017	0.345	0.660	0.125	0.35	0.852

#### Isotherm Study

The adsorption isotherms of composites, CS/MT and CS/MT/GO, for the binary system of organic dyes were investigated and are presented in Figure 16. The parameters fitted by the Langmuir and Freundlich models are shown in Tables 7 and 8. The *R* ^2^ values of CS/MT/GO (≥0.98) indicate that the Freundlich model better fits the Langmuir model for both RB5 (Figure 16a) and MB (Figure 16b) in binary systems. Compared to the binary system, the CS/MT/GO adsorbent showed higher Freundlich parameters (*K*_F_ and 1/*n*) in the single-dye system. In this case, *K*_F_ and 1/*n* were reduced in the presence of RB5 or MB. This suggests that organic pollutants compete for the same active sites on the adsorbent surface.^65^

**Figure 16:**
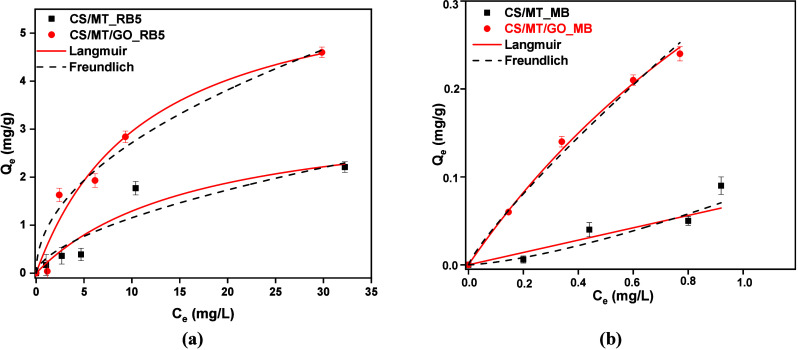
Isotherm study of CS/MT and CS/MT/GO in mix solution regarding (a) RB5 and (b) MB dyes.

**Table 7:** Langmuir Parameters of the Adsorption Process in a Binary System

Langmuir model				
pollutant	adsorbent	*Κ*_L_ (L/mg)	*Q*_m_ (mg/g)	*R* ^2^
RB5	CS/MT	0.060	3.45	0.898
	CS/MT/GO	0.087	6.32	0.955
MB	CS/MT	1.205	0.582	0.801
	CS/MT/GO	0.839	0.839	0.994

**Table 8:** Freundlich Parameters of the Adsorption Process in a Binary System

Freundlich model				
pollutant	adsorbent	*Κ*_F_ (mg/g) (L/mg)^1/*n*^	1/*n*	*R* ^2^
RB5	CS/MT	0.298	0.591	0.855
	CS/MT/GO	0.876	0.492	0.950
MB	CS/MT	0.079	1.410	0.848
	CS/MT/GO	0.315	0.556	0.988

#### Thermodynamic Analysis

The thermodynamic parameters of the binary systems of RB5 and MB organic dye were investigated. As shown in Table 9, the results indicated that the adsorption of organic pollutants from CS/MT and CS/MT/GO is a spontaneous process, as evidenced by the negative Δ*G* ^0^ value. Additionally, the Δ*H* ^0^ values are negative and confirm that the adsorption process is exothermic.

**Table 9:** Thermodynamic Parameters of the Adsorption Process in a Binary System

pollutant	*T* (K)	Δ*G* ^0^ (kJ/mol)	Δ*H* ^0^ (kJ/mol)	Δ*S* ^0^ (kJ mol^–1^ K^–1^)	ln *K*_c_
RB5	303	–3.08	–23.95	0.093	1.27
	313	–4.53			1.80
	323	–4.92			1.89
MB	303	–5.11	–5.39	0.001	2.10
	313	–5.48			2.18
	323	–5.98			1.95

### Real Wastewater Management

The adsorption study in real wastewater is essential because it determines how efficient the adsorbent material is under real conditions. In addition, wastewater contains a mixture of organic and inorganic compounds, which makes wastewater treatment more difficult. In this study, the physicochemical characteristics of the real textile wastewater used are pH 8.2 ± 0.1, chemical oxygen demand (COD) of 1035 ± 110 mg/L, total dissolved solids (TDS) of 860 ± 15 mg/L, turbidity of 125 ± 10 NTU, and conductivity of 9352 ± 125 μS/cm. Figure 17 presents the removal percentages of TDS from CS/MT and CS/MT/GO composites during wastewater treatment. After 24 h, CS/MT/GO exhibits higher removal efficiency than CS/MT due to the presence of GO in the polymeric matrix.

**Figure 17:**
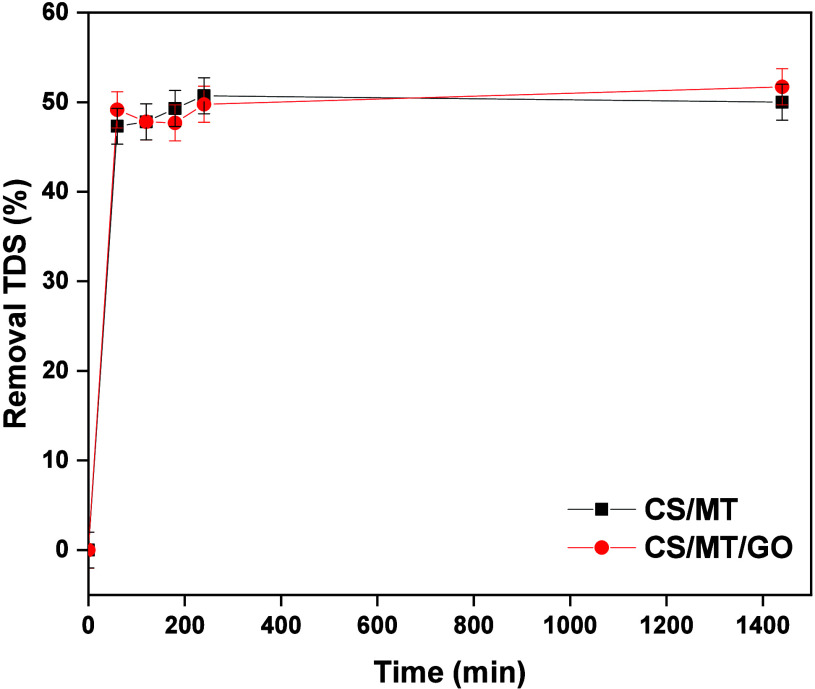
Removal efficiency of real textile wastewater from the CS/MT and CS/MT/GO composites.

As illustrated in Figure 18, the adsorption of real textile wastewater decreases as the contact time between the adsorbent material and real wastewater increases. Furthermore, CS/MT/GO (Figure 18b) exhibits a higher adsorption capacity than CS/MT (Figure 18a). Therefore, based on Figure 18c, the ultraviolet–visible (UV–vis) spectra before and after textile wastewater treatment are presented. After 24 h, the absorbance intensity appears to have decreased, demonstrating that the adsorbent composite is appropriate for dye removal from real wastewater and is suitable for industrial application.

**Figure 18:**
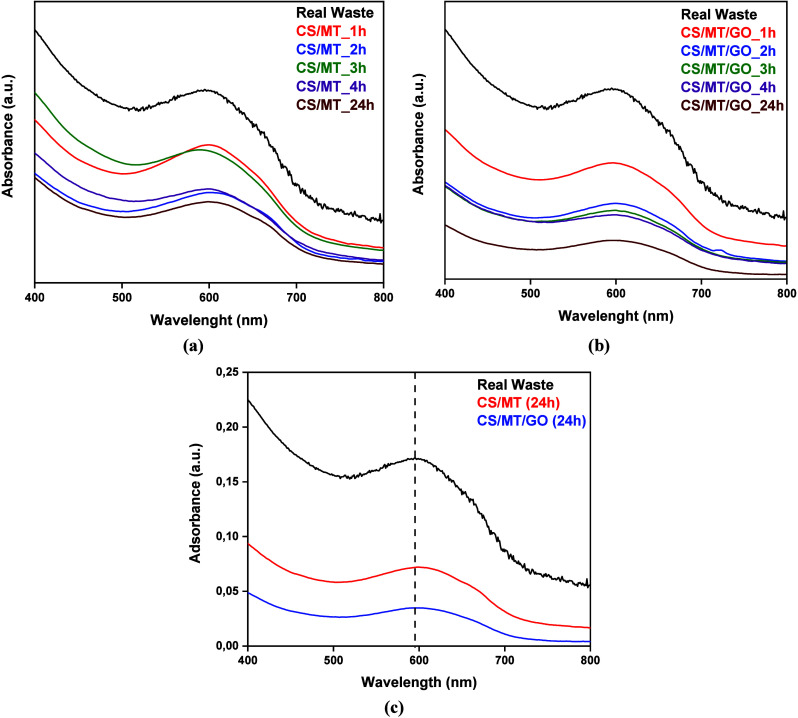
UV–vis spectrum of (a) CS/MT and (b) CS/MT/GO during real textile wastewater treatment, along with (c) comparative illustration of the composites after 24 h of treatment.

#### Comparison to Literature and Recommendations

The optimum adsorbent material CS/MT/GO is a highly suitable candidate for organic dye removal, as it demonstrated superior adsorption efficiency for both RB5 and MB compared to other adsorbents (Table 10). Specifically, CS/MT/GO achieved a removal efficiency of 99.1% at an initial dye concentration of 250 mg/L, along with the highest adsorption capacity (*Q*_m_ = 348.81 mg/g). According to the literature, other recent adsorbent materials, such as MWCNTs (*Q*_m_ = 231.84 mg/g), CCHB (*Q*_m_ = 169.49 mg/g), and PEI-CH (*Q*_m_ = 77.52 mg/g), have shown excellent removal efficiencies but lower adsorption capacities than CS/MT/GO. Additionally, the new composite exhibited outstanding performance in MB removal, surpassing other materials, such as charred *Parthenium* (*Q*_m_ = 89.40 mg/g) and Persian kaolin (*Q*_m_ = 29.85 mg/g). This enhanced performance is likely due to the synergistic combination of CS, MT, and GO, which forms a polymeric matrix with numerous active sites and a large surface area. In this study, the CS/MT/GO composite exhibits superior adsorption performance compared to conventional materials, achieving high efficiency under mild conditions. Its low-cost, eco-friendly composition and excellent reusability (90% capacity retention after 5 cycles) make it suitable for large-scale applications. Furthermore, the strong adsorption performance of CS/MT/GO in single and binary dye systems highlights its selectivity and practical relevance. These advantages underscore the composite’s innovation and potential for effective wastewater treatment.

**Table 10:** Comparative Analysis of the Dye Adsorption Performance of CS/MT/GO to Other Contiguous Adsorbents Found in the Literature

adsorbent	dye	*C*_0_ (mg/L)	dosage (g/L)	pH	removal (%)	*Q*_m_ (mg/g)	reference
CCHB^a^	RB5	100	1.0	3	98.0	169.49	^24^
PEI–CW^b^	RB5	50	0.1	3	93.0	77.52	^66^
MWCNTs^c^	RB5	25	1.0	3	86.5	231.84	^67^
CS/MTGO	RB5	250	1.0	2	99.1	348.81	this study
charred *Parthenium*	MB	25	0.5	7	93.4	89.40	^68^
Fe–BDC MOF^d^	MB	5	2.5	9	94.7	8.65	^69^
Persian kaolin	MB	10	2.0	7	90.0	29.85	^70^
CS/MTGO	MB	100	1.0	12	80.3	182.36	this study

a CS/pencil clay.

b Nano-ZnO/CS composite beads.

c Multi-walled carbon nanotubes.

d Fe–benzene dicarboxylic acid/terephthalic acid.

In addition, future research should aim to scale up the synthesis of CS/MT/GO composite beads and evaluate their performance in pilot-scale or continuous flow systems to better assess their real-world applicability. Moreover, long-term studies on the stability and regeneration efficiency over multiple regeneration cycles are essential for determining the durability of beads. In parallel, expanding the scope of tested pollutants and performing comprehensive environmental impact assessments, including life cycle analysis (LCA) of both the synthesis and disposal, will strengthen the case for the sustainability of the adsorbent. Furthermore, functional optimization of the composite, coupled with thorough economic evaluations and cost–benefit analyses, could significantly improve its viability for industrial wastewater treatment.

## Conclusion

In this research work, CS/MT/GO composite beads were successfully synthesized and evaluated for removal of both anionic (RB5) and cationic (MB) dyes. The composite exhibited a high adsorption capacity, reaching 348.81 mg/g for RB5 and 182.36 mg/g for MB and achieved removal efficiencies of 99.1 and 80.3%, respectively, under optimal conditions. Additionally, characterization confirmed enhanced surface morphology, pore size, and crystallinity. Furthermore, the adsorption followed a pseudo-second-order kinetic model and fitted well with the Freundlich isotherm, indicating multilayer chemisorption. The adsorption process was spontaneous, and the material showed good reusability, retaining 90% capacity for RB5 after 5 cycles. Overall, CS/MT/GO demonstrated a strong performance in single, binary, and real wastewater systems, highlighting its potential for practical industrial applications.

## Data Availability

Data will be made available on request.
